# Berberine: A Negentropic Modulator for Multi-System Coordination

**DOI:** 10.3390/ijms27020747

**Published:** 2026-01-12

**Authors:** Xiaolian Tian, Qingbo Chen, Yingying He, Yangyang Cheng, Mengyu Zhao, Yuanbin Li, Meng Yu, Jiandong Jiang, Lulu Wang

**Affiliations:** 1 State Key Laboratory of Bioactive Substance and Function of Natural Medicines, Institute of Medicinal Biotechnology, Chinese Academy of Medical Sciences & Peking Union Medical College, Beijing 100050, China; 18852072531@163.com (X.T.); chenqingbo14@163.com (Q.C.); heyingying212021@163.com (Y.H.); chengyangyang173@163.com (Y.C.); zhaomengyu85@163.com (M.Z.); yuanbinlii@163.com (Y.L.); yumeng@hightidetx.com (M.Y.); 2 Institute of Materia Medica, Chinese Academy of Medical Sciences & Peking Union Medical College, Beijing 100050, China

**Keywords:** Berberine, AMPK-centered mechanistic hub, combination strategies of BBR

## Abstract

Berberine (BBR), a protoberberine alkaloid with a long history of medicinal use, has consistently demonstrated benefits in glucose–lipid metabolism and inflammatory balance across both preclinical and human studies. These diverse effects are not mediated by a single molecular target but by BBR’s capacity to restore network coordination among metabolic, immune, and microbial systems. At the core of this regulation is an AMP-activated Protein Kinase (AMPK)-centered mechanistic hub, integrating signals from insulin and nutrient sensing, Sirtuin 1/3 (SIRT1/3)-mediated mitochondrial adaptation, and inflammatory pathways such as nuclear Factor Kappa-light-chain-enhancer of Activated B cells (NF-κB) and NOD-, LRR- and Pyrin Domain-containing Protein 3 (NLRP3). This hub is dynamically regulated by system-level inputs from the gut, mitochondria, and epigenome, which in turn strengthen intestinal barrier function, reshape microbial and bile-acid metabolites, improve redox balance, and potentially reverse the epigenetic imprint of metabolic stress. These interactions propagate through multi-organ axes, linking the gut, liver, adipose, and vascular systems, thus aligning local metabolic adjustments with systemic homeostasis. Within this framework, BBR functions as a negentropic modulator, reducing metabolic entropy by fostering a coordinated balance among these interconnected systems, thereby restoring physiological order. Combination strategies, such as pairing BBR with metformin, Sodium-Glucose Cotransporter 2 (SGLT2) inhibitors, and agents targeting the microbiome or inflammation, have shown enhanced efficacy and substantial translational potential. Berberine ursodeoxycholate (HTD1801), an ionic-salt derivative of BBR currently in Phase III trials and directly compared with dapagliflozin, exemplifies the therapeutic promise of such approaches. Within the hub–axis paradigm, BBR emerges as a systems-level modulator that recouples energy, immune, and microbial circuits to drive multi-organ remodeling.

## 1. Introduction

**Figure 1:**
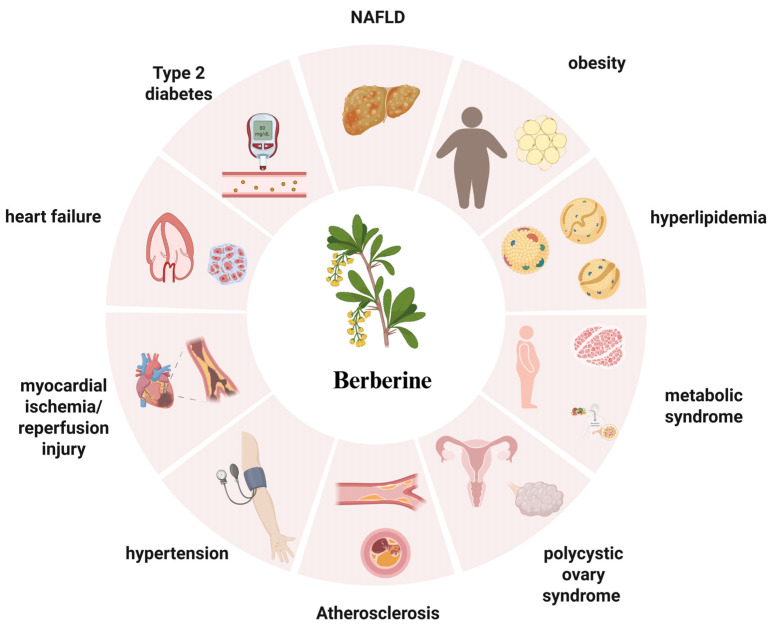
BBR demonstrates therapeutic effects on cardiometabolic conditions, including metabolic diseases such as type 2 diabetes, obesity, non-alcoholic fatty liver disease, hyperlipidemia, metabolic syndrome, and polycystic ovary syndrome (PCOS), as well as cardiovascular diseases like atherosclerosis, hypertension, myocardial ischemia/reperfusion injury, and heart failure. Created in BioRender. Tian, X. (2025) https://BioRender.com/ud9id3s, accessed on 12 December 2025.

Evidence from both preclinical and clinical studies consistently demonstrates that berberine (BBR) provides wide-ranging benefits across the cardiometabolic disease spectrum (Figure 1). Cardiometabolic diseases are intrinsically multifaceted, arising from interconnected disturbances in energy homeostasis, inflammation, and vascular dysfunction [1,2]. While conventional single-target therapies have yielded only partial benefits, BBR—a protoberberine alkaloid with a long history of medicinal use—exemplifies a multi-dimensional systems-level modulator.

In metabolic disorders, BBR improves glycemic control in type 2 diabetes, reduces circulating triglycerides and cholesterol in hyperlipidemia, and alleviates metabolic dysfunction-associated steatotic liver disease (MASLD) by decreasing hepatic steatosis and modulating lipid flux [3,4]. These metabolic effects extend robustly to vascular protection: BBR mitigates atherosclerosis progression, lowers blood pressure, and preserves endothelial function, largely through AMPK activation, lipid normalization, and anti-inflammatory actions [5].

These pleiotropic benefits suggest that BBR operates not through a single target, but by coordinating the modulation of energy-sensing, lipid-handling, and vascular-inflammatory pathways. However, previous summaries have often described BBR’s pharmacology as a series of discrete, parallel effects—such as improving gut microbiota [6], attenuating inflammatory mediators [7], enhancing insulin sensitivity [8], and repairing mitochondrial dysfunction [9]—without clarifying how these processes interact. While this compartmentalized view captures the breadth of BBR’s actions, it obscures their network-level integration, a critical gap in understanding its efficacy against cardiometabolic multimorbidity.

To bridge this gap, this study proposes a systems-level framework centered on an AMPK-anchored metabolic hub. AMPK serves as the primary energy-sensing integrator, coordinating crosstalk with key pathways including InsR–PI3K–AKT–mTOR, SIRT1/3, NF-κB, and NLRP3 [10,11]. Through upstream energy and redox cues, this hub governs fundamental processes such as glucose uptake, lipid metabolism, and inflammatory responses. AMPK enhances glucose utilization via GLUT4 translocation (through the AKT–AS160/TBC1D4 and AMPK–TBC1D1 routes), suppresses de novo lipogenesis (by phosphorylating ACC and inhibiting SREBP-1c), promotes fatty acid oxidation (via CPT1 activation), and attenuates inflammatory signaling. Reciprocal regulation between AMPK and the InsR–PI3K–AKT–mTOR axis dynamically balances anabolic and catabolic processes, while SIRT1/3 reinforces AMPK signaling through the deacetylation of LKB1 and PGC-1α, promoting mitochondrial biogenesis and redox adaptation [12,13].

Crucially, this hub is dynamically regulated by system-level inputs. Signals from the gut, mitochondria, and epigenome converge to modulate its activity. BBR enhances intestinal barrier integrity and modulates microbial metabolites—such as short-chain fatty acids (SCFAs) and secondary bile acids—that activate TLR4, FXR, and TGR5 [14]. It also improves mitochondrial respiration and redox balance, interacting with epigenetic regulators that can encode or reverse metabolic memory [15]. Collectively, these inputs establish a self-reinforcing gut–liver–adipose axis, providing a mechanistic basis for the durability and cross-indication efficacy observed in clinical studies. Within this framework, BBR acts as a negentropy modulator, reducing metabolic entropy by promoting a coordinated balance among these interconnected systems, thus restoring overall physiological order.

This perspective reframes BBR as a network modulator rather than a single-target drug. It rationalizes its pleiotropic and durable benefits, supports the design of mechanism-based combination strategies—including metformin, SGLT2 inhibitors, and microbiome-directed strategies—and motivates the use of network-level and organ-axis endpoints (such as cardiometabolic biomarkers, bile-acid signaling, and microbial or inflammatory readouts) for assessing therapeutic effects. HTD1801, an ionic-salt derivative of BBR currently in Phase III trials, exemplifies the translational promise of such approaches.

The following sections delineate the architecture of the AMPK-centered metabolic hub, explore its upstream regulators and downstream effectors across organ systems, and discuss combination strategies that leverage this network to enhance therapeutic precision in cardiometabolic disease.

## 2. “Multi-Targeting—System-Wide Remodeling” Pharmacological Profile

### 2.1. Integrated Signaling Hub

**Figure 2:**
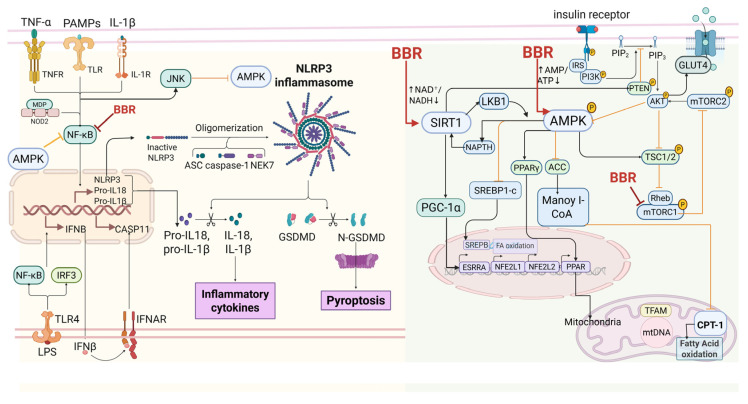
BBR targets core hubs such as AMPK, SIRT1, and NF-κB. By activating AMPK, it enhances insulin sensitivity and GLUT4 translocation, thereby lowering blood glucose levels. Simultaneously, BBR activates SIRT1, with AMPK and SIRT1 mutually reinforcing each other. Through transcriptional regulation, including deacetylation and mitochondrial programming, BBR further amplifies its regulatory effects and modulates the PI3K/AKT/mTOR pathway. Inhibiting NF-κB not only activates AMPK but also inhibits NLRP3, exerting anti-inflammatory effects. In this figure, “↑” denotes activation, “↓” denotes reduction and “⊣” denotes inhibition. Created in BioRender. Tian, X. (2025) https://BioRender.com/7hnaic1, accessed on 12 December 2025.

The pleiotropic effects of BBR are best understood not as discrete actions on separate targets, but as the outcome of its modulation of an integrated metabolic signaling hub centered on AMPK. This hub functions as a proximal controller, dynamically integrating diverse upstream inputs to drive coherent metabolic reprogramming (Figure 2).

At its core, AMPK activation—driven by energy and redox sensing—orchestrates context-dependent crosstalk with key signaling networks. It coordinates with the insulin receptor (InsR)–PI3K–AKT axis to regulate shared downstream processes such as GLUT4 translocation and glucose utilization. Concurrently, it suppresses de novo lipogenesis through ACC phosphorylation and SREBP-1c inhibition. This metabolic regulation is reinforced by interactions with SIRT1/3, which enhance transcriptional regulation and mitochondrial programming via deacetylation. Furthermore, inflammatory (NF-κB, NLRP3) and growth-signal (PI3K–AKT–mTOR) pathways are woven into this network, providing critical contingencies that modulate metabolic set-points in response to physiological and pathological cues [16].

#### 2.1.1. AMPK Pathway: The Central Energy-Sensing Node

Efficient regulation of cellular energy status is fundamental to metabolic homeostasis. AMPK, a heterotrimeric complex (α catalytic subunit; β/γ regulatory subunits), is allosterically activated by increased AMP/ADP:ATP ratios and fully activated by Thr172 phosphorylation via LKB1 (STK11), CaMKKβ, or TAK1 signaling pathways [17,18]. Mechanistic studies suggest that BBR transiently inhibits mitochondrial complex I, elevates AMP:ATP ratios, and thereby activates AMPK to restore metabolic balance [19,20]. Furthermore, some studies indicate that BBR sustains AMPK activity by downregulating UHRF1, which interacts with and inhibits AMPKα1 [21,22]. AMPK and SIRT1 form a reciprocal self-reinforcing loop: AMPK upregulates NAMPT to increase intracellular NAD^+^ levels, stimulating SIRT1 activity, while SIRT1 deacetylates LKB1, reinforcing AMPK activation and maintaining energy/redox homeostasis [23].

#### 2.1.2. Sirtuin Signaling: Epigenetic and Metabolic Integration

Sirtuins (SIRT1–SIRT7) are NAD^+^-dependent deacetylases with distinct subcellular distributions and functions [24,25]. BBR enhances SIRT1-linked pathways involved in insulin sensitivity, inflammation control, and mitochondrial function. It improves adipose and hepatic metabolism through the AMPK–SIRT1 axis, alleviates β-cell dysfunction via the miR-204/SIRT1 pathway, and mitigates cardiac mitochondrial aging in experimental models. The AMPK–SIRT1–PGC-1α triad operates as a redox-responsive module that coordinates mitochondrial biogenesis, fatty-acid oxidation, and adaptive remodeling [26,27,28]. BBR, as a mitochondria-targeting agent, selectively and reversibly dissociates mitochondrial CI through SIRT3-dependent NDUFS1 deacetylation to improve hepatocellular glucose and lipid metabolism [29].

#### 2.1.3. NF-κB and NLRP3 Inflammasome: Inflammatory Gatekeepers

NF-κB family members (p50, p52, p65/RelA, c-Rel, and RelB) orchestrate inflammatory and survival responses. Under resting conditions, IκB proteins sequester NF-κB in the cytosol; activation triggers IκB phosphorylation and degradation, enabling nuclear translocation [30]. BBR reduces IκB phosphorylation and NF-κB nuclear localization, while SIRT1-mediated p65 deacetylation further suppresses inflammatory transcription. BBR also dampens NLRP3 inflammasome activation, limiting IL-1β/IL-18 maturation and shifting macrophage polarization from the pro-inflammatory M1 to the anti-inflammatory M2 phenotype [31,32]. A potential direct interaction between BBR and EIF2AK2 (PKR) has been proposed, selectively modulating JNK, NF-κB, AKT, and NLRP3 signaling, though this requires independent confirmation [33].

#### 2.1.4. PI3K–Akt/mTOR Axis: Context-Dependent Modulator

The PI3K–AKT–mTOR axis integrates anabolic and growth cues with energy status. BBR exerts context-dependent effects on this pathway: in insulin-resistant states, it enhances AKT phosphorylation and glucose uptake, whereas under nutrient-rich or oncogenic conditions, it can suppress mTORC1 signaling through TSC2 and Raptor phosphorylation by AMPK [34,35]. This dual modulation harmonizes anabolic–catabolic balance and links growth signaling to energy availability, providing a mechanistic basis for BBR’s tissue-specific actions across metabolic and neoplastic settings [36].

#### 2.1.5. Additional Regulatory Nodes

Beyond these core hubs, BBR influences additional metabolic circuits. It modulates adipogenesis through C/EBPα and PPARγ, promotes lipid handling via AMPK–SIRT1–PPAR programs, and upregulates LDLR expression through an ERK-dependent mechanism, contributing to improved lipid clearance and cardioprotection. As a complex I modulator, BBR adjusts the ATP/NADH balance and supports anti-oxidative and anti-inflammatory responses [8,16,37]. Collectively, these interconnected actions highlight AMPK as the organizing node through which BBR orchestrates system-wide metabolic remodeling.

### 2.2. Upstream Drivers of the Hub

**Figure 3:**
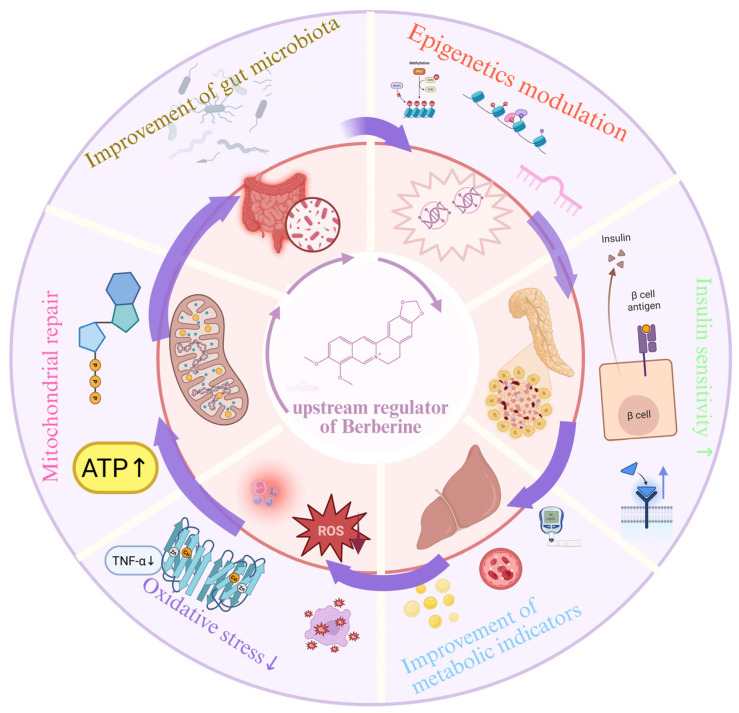
BBR is mainly absorbed by the intestine and acts on gut bacteria, epigenetics modulation, and mitochondrial energy. It can repair the gut microbiota, thereby the gut microbiota enter the circulatory system through itself and its metabolites (such as dihydroberberine) and then affect the epigenetic regulation of host cells (especially liver, adipose tissue, immune cells, etc.). This effect on epigenetic regulation improves insulin sensitivity, further enhancing metabolic markers and reducing free radical production and repairing mitochondrial damage. Mitochondrial repair further optimizes the intestinal environment, creating a closed loop of improvement. In this figure, “↑” denotes incrassation, and “↓” denotes reduction. Created in BioRender. Tian, X. (2025) https://BioRender.com/cgqqqct, accessed on 12 December 2025.

BBR’s systemic effects are not mediated by directly targeting the signaling hub alone; it profoundly modulates upstream drivers that continuously feed into and fine-tune hub activity. This establishes a “Hub and Spoke” model, where signals originating from the gut microbiome, epigenetic programs, mitochondrial energetics, and systemic immune-inflammatory tone converge on the central AMPK-InsR hub. Together, these inputs form a multi-dimensional control system that calibrates metabolic signaling and determines the persistence of therapeutic remodeling (Figure 3).

#### 2.2.1. Microecological Regulation and Metabolic-Axis Repair

A key upstream driver is the bidirectional modulation of the gut microbiota–host metabolism axis. At the compositional level, BBR enriches beneficial taxa, such as Akkermansia and Bifidobacterium, while suppressing Gram-negative and TMA-producing bacteria. Akkermansia strengthens the mucus layer (via MUC2), limiting endotoxin translocation and metabolic inflammation, while Bifidobacterium increases SCFAs, particularly butyrate, which stimulates GLP-1 secretion and improves insulin sensitivity. Suppressing Gram-negative bacteria reduces LPS production and decreased TMA formation lowers circulating TMAO, an atherogenic metabolite [38,39,40,41].

Beyond microbial composition, BBR remodels microbial metabolites. Butyrate induces GLP-1 release; modified secondary bile acids (e.g., reduced deoxycholic acid) signal through receptors like TGR5; and modulated indole derivatives contribute to anti-inflammatory signaling and barrier repair [42,43,44]. BBR directly enhances intestinal barrier integrity by upregulating tight-junction proteins (e.g., zonula occludens-1 and occludin) and activating enterocyte AMPK to boost mucin secretion, thereby restricting pathogen access and reducing innate immune activation [42,45].

Collectively, these actions stabilize the gut–liver interface, reduce the systemic endotoxin and inflammatory tone, and restore the metabolic–immune equilibrium.

#### 2.2.2. Epigenetics Modulation and Reversal of “Metabolic Memory”

Building on inter-organ communication, BBR directly engages epigenetic mechanisms to counteract metabolic memory—the persistent maladaptive transcriptional programs formed during prior metabolic stress [46,47]. This reprogramming resets cellular identity toward metabolic flexibility and redox resilience across several layers.

1. DNA Methylation

BBR inhibits DNMT1/3a activity at insulin-signaling loci (INSR, GLUT4), leading to promoter demethylation and restored insulin sensitivity. At adipogenic genes (e.g., PPARγ), BBR induces TET2-linked demethylation, reducing lipid accumulation [48,49,50]. Additional evidence shows decreased methylation at gluconeogenic promoters, resulting in reduced hepatic glucose output and improved fasting glycemia [51,52].

2. Histone Modification

Through SIRT1-dependent deacetylation and the inhibition of HDAC3/4, BBR modulates histone acetylation and methylation at metabolic and oxidative-stress genes. This includes increased H3K9ac at fatty-acid oxidation genes (CPT1A) and removal of repressive marks such as H3K27me3 at antioxidant loci. Chromatin remodeling at inflammatory promoters further limits NF-κB-driven transcription [53,54,55].

3. Non-Coding RNAs

BBR downregulates key miRNAs involved in metabolic dysfunction: reducing miR-34a (de-repressing SIRT1 and supporting mitochondrial biogenesis); lowering miR-122 (limiting lipogenic enzyme expression and hepatic steatosis); and decreasing miR-21 (de-repressing PTEN and enhancing AKT-dependent GLUT4 trafficking) [53]. Across these layers—DNA, histone, and miRNA—BBR resets the epigenome toward metabolic flexibility and redox resilience. Future directions include translational validation through target-tissue epigenomic profiling in defined responder subtypes.

#### 2.2.3. Mitochondrial Energy and Redox Sensing

Mitochondria serve as both targets and critical upstream sensors for BBR. BBR directly interacts with mitochondrial energy programs, elevating the AMP/ATP ratio, activating AMPK, and alleviating insulin signaling inhibition. It promotes PGC-1α-mediated mitochondrial biogenesis, enhances hepatic fatty-acid oxidation, and reduces oxidative stress, thereby restoring redox homeostasis and sensitizing the hub to endocrine and nutrient signals [9,56]. Through immune–inflammatory signaling, BBR suppresses NF-κB activation, restrains NLRP3 inflammasome assembly, and reduces systemic inflammation, alleviating upstream drivers of insulin resistance and metabolic stress [57]. This immune gating reduces signal noise at the hub and expands its dynamic range for metabolic control [7,33,58].

#### 2.2.4. Systemic Feedback Linking Upstream Drivers to the AMPK Hub

These diverse upstream inputs do not operate in isolation, they converge to establish a self-reinforcing, multi-organ circuit (e.g., gut–liver–adipose axis). For instance, gut-derived signals (SCFAs, bile acids) reduce endotoxin burden and inflammation, which improves insulin sensitivity. Enhanced AMPK–InsR signaling, in turn, decreases oxidative stress and supports mitochondrial repair. Restored mitochondrial function then feeds back through bile-acid and redox signaling to further stabilize the gut barrier and microbial ecology.

Notably, this loop can be initiated from multiple entry points—microbial metabolites, mitochondrial energy cues, epigenetic programs, or inflammatory triggers—all converging on the AMPK-InsR hub. This network perspective offers a mechanistic rationale for BBR’s sustained efficacy beyond transient biomarker changes, supporting the use of integrative clinical endpoints (e.g., MRI-PDFF, glycemic indices, lipid and inflammatory markers) that reflect hub- and axis-level responses.

### 2.3. Organ-Axis Remodeling: From Local Repair to Systemic Homeostasis

**Figure 4:**
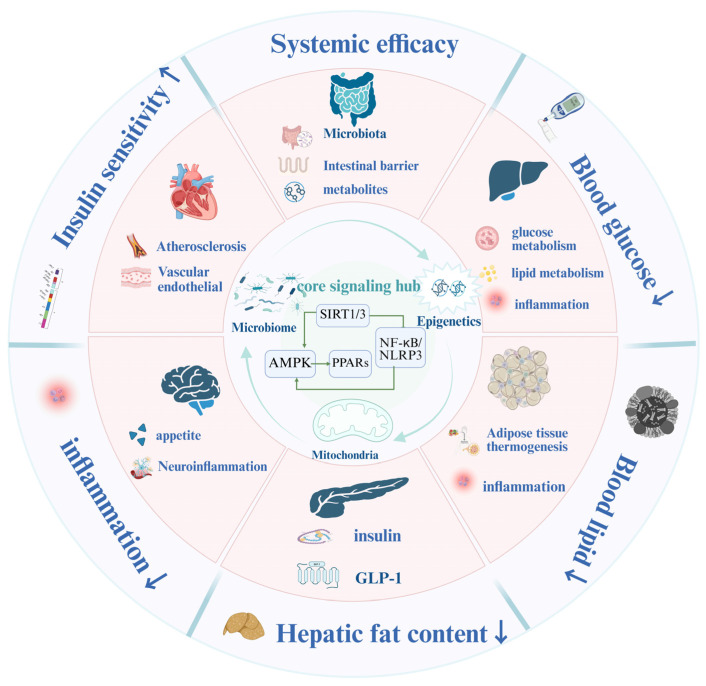
BBR uses the gut microbiota and epigenetics as upstream regulatory sources. By influencing the core hub centered on AMPK-InsR, BBR regulates the homeostasis of the gut, liver, pancreas, adipose tissue, brain, and cardiovascular system, thereby producing systemic effects such as the reduction of blood glucose and lipids, anti-inflammation, and an increase in insulin sensitivity. In this figure, “↑” denotes incrassation, and “↓” denotes reduction. Created in BioRender. Tian, X. (2026) https://BioRender.com/h1x80t1, accessed on 3 January 2026.

The integrated signaling hub and its upstream drivers ultimately translate their effects through organ-axis remodeling. BBR acts across key metabolic organs—including the liver, intestine, adipose tissue, pancreas, cardiovascular system, and brain—producing coordinated improvements in insulin sensitivity, lipid handling, and energy metabolism (Figure 4). These organ-level actions collectively extend the influence of the AMPK-centered hub to the systemic level.

#### 2.3.1. Organ-Level Actions: From Local Repair to Systemic Benefit

The therapeutic effects of BBR are rooted in its direct action on individual organs, which serve as the foundational units of systemic remodeling.

Liver: BBR enhances hepatic fatty-acid β-oxidation through PPARα/CPT1 and suppresses de novo lipogenesis via SREBP-1c inhibition [59]. It improves insulin signaling by increasing InsR expression and repressing gluconeogenic genes (G6PC, PCK1), while attenuating fibrosis through the inhibition of TGF-β/Smad signaling and reducing collagen deposition.

Pancreas: BBR exerts anti-fibrotic and β-cell-protective effects through the modulation of TGF-β/Smad and oxidative-stress pathways [60]. In preclinical and limited clinical studies, BBR increases GLP-1 bioactivity—via stimulation of L-cell secretion and potential inhibition of intestinal DPP-4—thereby enhancing insulin release and reducing β-cell stress [61].

Intestine: BBR restores epithelial barrier integrity by upregulating ZO-1 and occludin, decreasing paracellular permeability, and suppressing TLR4/MyD88 signaling. These effects reduce dysbiosis-driven inflammation and prevent systemic endotoxin leakage [44].

Adipose Tissue: BBR promotes thermogenic remodeling via UCP1 activation, facilitates white-to-beige/brown adipose tissue transition, and reduces lipid accumulation [62]. It also reprograms immune tone in adipose tissue—shifting macrophages from pro-inflammatory M1 to anti-inflammatory M2 phenotypes—and lowers resistin levels, alleviating adipose inflammation and insulin resistance.

Cardiovascular System: BBR enhances endothelial nitric oxide synthase (eNOS) activity and NO bioavailability, contributing to vasodilation and reduced platelet aggregation. It also exhibits anti-atherogenic effects by downregulating LOX-1, limiting oxidized LDL uptake, and upregulating ABCA1 to promote reverse cholesterol transport [7,63].

Brain: BBR supports neuro-metabolic communication, limits Aβ/Tau aggregation, increases BDNF expression, and mitigates neuroinflammation, partly through gut–brain axis signaling mediated by SCFAs and reduced microglial TLR4 activation [62,64,65].

#### 2.3.2. Inter-Organ Axis Communication: Orchestrating Systemic Harmony

Beyond discrete organ effects, BBR’s true therapeutic power lies in its ability to orchestrate axis-level communication across interconnected metabolic circuits, primarily originating from the restored gut ecosystem.

Gut–Liver Axis: Microbial metabolites, such as SCFAs, activate FFAR2/3 receptors to suppress SREBP-1c-driven lipogenesis. Simultaneously, BBR activates hepatic AMPK–SIRT1, upregulates PPARα, and accelerates fatty acid oxidation, reinforcing hepatic energy efficiency.

Gut–Pancreas Axis: BBR elevates GLP-1 levels both directly, via SCFA stimulation of L-cells, and indirectly through reduced intestinal DPP-4 activity in preclinical models. Enhanced GLP-1 signaling boosts insulin secretion and protects β-cells from metabolic stress [39].

Gut–Adipose Axis: Microbial butyrate and related SCFAs activate PPARγ and UCP1-dependent thermogenesis, while reduced endotoxemia lowers macrophage infiltration and inflammatory signaling in adipose tissue [52,66].

Gut–Brain Axis: SCFAs and vagal signaling enhance hypothalamic PYY release and reduce ghrelin, curbing food intake and promoting energy balance. Simultaneously, diminished microbial endotoxins reduce microglial TLR4 activation, limiting neuroinflammation and contributing to cognitive resilience in metabolic and neurodegenerative models [42,44].

#### 2.3.3. Systemic Integration: From Coordinated Networks to Clinical Resilience

These hierarchically coupled organ and axis-level effects form an integrated physiological network. Through this network, BBR restores system-wide metabolic–inflammatory homeostasis. It transforms the AMPK hub from a cellular energy sensor into a systemic coordinator, aligning local energy sensing with inter-organ communication. This paradigm explains how BBR achieves durable, multi-organ benefits in cardiometabolic diseases—not by overpowering a single pathway, but by re-tuning the entire system’s capacity for self-regulation and resilience. This system-level understanding directly informs the rational design of combination therapies and the selection of network-based clinical endpoints, as discussed in the following section.

## 3. Systematic Exploration of Drug Combinations to Amplify BBR’s Therapeutic Network

The therapeutic frontier for complex cardiometabolic diseases is increasingly defined by rational drug combinations that target disease networks, moving beyond single-pathway modulation. Building on the framework established in previous sections—which defines BBR as an AMPK-centered metabolic hub integrated with multi-organ signaling axes—this section explores how combination therapies can be designed to enhance the robustness and clinical efficacy of this therapeutic network.

Combination therapy is a cornerstone in the treatment of complex diseases such as HIV and tuberculosis, where it overcomes resistance and amplifies efficacy [66,67]. A similar systems-based logic is now essential for metabolic disorders. For BBR, the most promising combinations are those that engage complementary pathways around the AMPK–SIRT1/3–NF-κB core, reinforce coordination along the gut–liver–pancreas–adipose–brain axes, optimize pharmacokinetic exposure where needed, and maintain favorable safety and tolerability [68,69].

**Figure 5:**
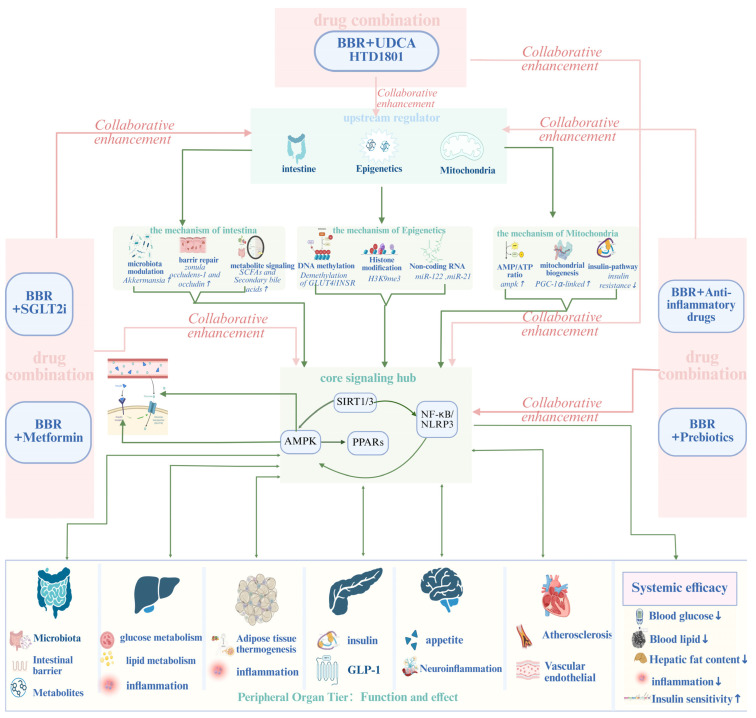
The combination of BBR with the five drugs shown in the figure can synergistically enhance upstream regulatory sources, including gut microbiota (increasing the species abundance of gut microbiota, repairing the gut barrier, increasing the production of metabolites, etc.) and epigenetics (DNA methylation, histone modification, regulation of non-coding RNAs, etc.). Meanwhile, it can also synergistically regulate signaling pathways, such as the SIRT1 and NLRP3 signaling pathways centered around AMPK-InsR. In this figure, “↑” denotes incrassation, and “↓” denotes reduction. Created in BioRender. Tian, X. (2026) https://BioRender.com/93mnik1, accessed on 3 January 2026.

Among the various approaches explored, four categories illustrate distinct but converging strategies to amplify BBR’s network pharmacology and therapeutic durability. The first involves ionic-salt integration with ursodeoxycholic acid (UDCA), forming HTD1801, a supramolecular entity that couples bile-acid signaling with AMPK activation. The second strategy leverages co-administration with metformin, aligning two energy-sensing activators that act through different upstream routes to converge on the same metabolic hub. The third strategy involves pairing BBR with SGLT2 inhibitors, combining renal glucose excretion with BBR’s insulin-sensitizing and anti-inflammatory actions to address the cardio–renal–metabolic continuum. Lastly, microbiome- and inflammation-directed strategies—such as prebiotics, probiotics, and anti-inflammatory agents—reinforce gut-derived signaling and mitigate systemic inflammatory noise. Collectively, these strategies demonstrate how BBR can serve not only as an active compound but also as a platform for rational combination design, translating mechanistic complementarity into system-wide therapeutic coherence (Figure 5, Table 1).

### 3.1. BBR + UDCA (HTD1801)

This strategy exemplifies molecular integration, creating a novel chemical entity that merges complementary pharmacophores. UDCA is a clinically established bile acid used in treating cholestatic liver diseases and related metabolic conditions. Its mechanisms of action include anti-inflammatory, anti-oxidative, and cytoprotective effects [82]. In supramolecular formulations, BBR–UDCA nanoparticles have demonstrated efficacy in inflammatory bowel disease models, improving mucosal inflammation and barrier function [83]. HTD1801 (BBR ursodeoxycholate, BUDCA) is a novel molecular entity (NME)—an equimolar ionic salt of BBR and UDCA—designed as an enteric–hepatic metabolic regulator. Despite being equimolar, pharmacokinetic studies show significantly higher BBR exposure, indicating differentiated distribution and complementary sites of action [84].

Mechanistically, HTD1801 activates AMPK, suppresses the NLRP3 inflammasome, enhances insulin receptor (InsR) and LDL receptor (LDLR) signaling, and modulates bile-acid receptors (FXR/TGR5), thereby orchestrating glucose, lipid, and inflammatory regulation [70,71]. Clinically, it has demonstrated superior improvements in glycemia, insulin sensitivity, hepatic fat content (MASLD/MASH), and liver enzymes (ALT/AST) compared to either component alone, particularly in T2DM and fatty liver disease cohorts. This validates the therapeutic potential of co-targeting metabolic and bile-acid pathways [70,71,83].

### 3.2. BBR + Metformin

This combination leverages convergent pharmacology at the AMPK hub via distinct upstream triggers. Both BBR and metformin are widely used in the management of T2D and metabolic syndrome [74,85]. They converge on AMPK through distinct upstream triggers: metformin suppresses hepatic gluconeogenesis and enhances peripheral glucose uptake/utilization, improving insulin sensitivity and inhibiting lipogenesis [86,87,88], while BBR typically acts via mitochondrial complex I and energy/redox stress, increasing the AMP/ATP ratio and indirectly activating AMPK. The different initiation points and tissue time-courses suggest potential additivity or synergy at the AMPK hub. Both agents also modulate the gut microbiome and the GLP-1 axis (with microbiome effects well established for metformin, while intestinal DPP-4 modulation by BBR remains primarily preclinical), supporting barrier integrity, SCFAs, and incretin biology [88,89,90]. In combination, these agents have been reported to improve glycemia, lipids, insulin sensitivity, and inflammatory tone beyond the effects of monotherapy [87,91].

### 3.3. BBR + Prebiotics

This approach employs a sequential, synergistic strategy to optimize the gut microenvironment. Prebiotics (e.g., FOS, inulin) are selectively utilized by beneficial gut bacteria, increasing SCFAs and improving barrier function, immune response, and energy metabolism [92,93]. BBR has been shown to suppress conditional pathobionts (e.g., Enterobacteriaceae) while enriching beneficial taxa such as Akkermansia and Bifidobacterium [94]. Co-administration with prebiotics can amplify these effects by providing substrates for BBR-enriched taxa, thereby boosting SCFAs, stabilizing the microbiome, and enhancing GLP-1 secretion. Prebiotics may also mitigate potential off-target suppression of commensals by BBR, preserving microbiome diversity. Clinical and preclinical studies suggest synergistic or additive improvements in blood glucose, lipids, obesity traits, and low-grade inflammation [95,96].

### 3.4. BBR + Anti-Inflammatory Drugs

This strategy achieves multi-layer inhibition within the inflammatory cascade. BBR and conventional anti-inflammatory agents act at complementary nodes within the same inflammatory network, providing a mechanistic basis for synergy [97,98]. BBR inhibits NF-κB signaling (e.g., by limiting IKK activity, IκB degradation, and p65 nuclear translocation), modulates MAPK (ERK/JNK/p38) pathways, NLRP3 inflammasome assembly and activation, and JAK/STAT signaling, thereby reducing upstream signal propagation and pro-inflammatory gene transcription. Nonsteroidal anti-inflammatory drugs (NSAIDs) lower prostaglandin production through cyclooxygenase (COX) inhibition, with COX-2 itself being an NF-κB target gene, thus attenuating downstream effector mediators [99]. Glucocorticoids bind to cytoplasmic glucocorticoid receptors, translocate to the nucleus, repress NF-κB-driven transcription, induce anti-inflammatory genes (e.g., IκBα), and also interact with MAPK signaling [100,101]. In aggregate, BBR tends to suppress upstream signaling and network crosstalk, while NSAIDs and glucocorticoids inhibit effector synthesis and nuclear transcription, respectively. Concurrent targeting of these layers often provides superior inhibition compared to monotherapy. Beyond pathway crosstalk, BBR’s modulation of immune cell activation, oxidative stress, gut ecology, and metabolism may enable dose-sparing of anti-inflammatory drugs. As proof-of-concept, carrier-free nanostructures (e.g., BBR + magnolol) have demonstrated robust cytokine control, colonic barrier preservation, and microbiota modulation in dextran sulfate sodium-induced colitis models [102].

### 3.5. BBR + SGLT2 Inhibitors

This pairing represents a multi-organ complementary strategy, addressing the cardio–renal metabolic continuum through distinct yet synergistic mechanisms.

Glycaemic Control: SGLT2 inhibitors lower plasma glucose independently of insulin by inhibiting renal proximal-tubule SGLT2, reducing glucose reabsorption, and promoting glucosuria [103,104]. BBR improves glycemia through insulin-sensitizing effects, modulation of intestinal glucose handling and incretin biology (GLP-1), and AMPK-linked energy/redox signaling. These mechanisms, being independent yet complementary, make co-administration potentially additive or even synergistic in lowering glucose, particularly in T2D with significant insulin resistance and/or β-cell dysfunction.

Metabolic-Syndrome Endpoints: SGLT2 inhibitors promote caloric loss and induce osmotic diuresis/natriuresis, aiding weight reduction and lowering blood pressure. Their effects on lipids are generally neutral to modestly favorable and are variable across agents [105]. BBR consistently improves TC, LDL-C, and triglycerides (with small increases in HDL-C), and—via AMPK/mTOR and related pathways—suppresses hepatic lipogenesis while enhancing fatty-acid oxidation, benefiting MASLD/NAFLD features. The combination may lead to broader improvements in weight, blood pressure, lipids, and hepatic fat.

Cardio-Renal Protection: SGLT2 inhibitors reduce preload/afterload (via diuresis and natriuresis), attenuate glomerular hyperfiltration through tubuloglomerular feedback, improve myocardial energetics (increased ketone utilization), and suppress fibrosis and inflammation through pathways such as TGF-β/Smad, alongside reductions in oxidative stress [105,106,107]. BBR complements these effects with anti-inflammatory, anti-oxidative, and anti-fibrotic actions, improves endothelial function, modulates autophagy, and exhibits anti-atherosclerotic effects. Together, these profiles may more effectively counter structural remodeling in the heart and kidneys while addressing the metabolic drivers of cardio-renal disease.

Microbiome-linked synergy (hypothesis generating): Preclinical and exploratory clinical data suggest SGLT2 inhibitors may alter gut microbiota composition (e.g., increasing butyrate-producing taxa), though consistency and clinical relevance remain under investigation [108]. The BBR–SGLT2i combination could, via distinct pathways, jointly enhance gut ecology and barrier integrity, reduce “leaky-gut”-associated low-grade inflammation, and subsequently improve insulin sensitivity while mitigating cardio-renal risk.

### 3.6. System-Level Interpretation

Whether through molecular fusion (HTD1801), parallel hub activation (metformin), ecological reinforcement (prebiotics), layered pathway inhibition (anti-inflammatories), or multi-organ task-sharing (SGLT2 inhibitors), these approaches all enhance the robustness and adaptive capacity of the therapeutic network. They exemplify “multi-target coherence”—a rational design philosophy where individual drugs are chosen not merely for additive effects, but for their ability to collaboratively restore the dynamics of a dysfunctional physiological network.

This system-level perspective provides a powerful framework for future combination therapy design. It moves beyond empirical pairing towards mechanism-guided precision, prioritizing agents that complement BBR’s role as a system-level modulator. It also argues for the use of network-sensitive clinical endpoints (e.g., composite scores of metabolic, inflammatory, and microbial health) to capture the true therapeutic impact of such synergistic regimens.

## 4. Discussion

BBR, a natural alkaloid with numerous biological activities, has shown great promise as a therapeutic agent for various diseases, including metabolic disorders, cardiovascular diseases, and cancer. The therapeutic action of BBR is rooted in a coherent biological network rather than a single target. Converging evidence supports its role as a multi-pathway integrator centered on the AMPK–SIRT1–PGC-1α axis, with regulatory inputs from the gut microbiota–immune interface, mitochondrial energetics, epigenetic programs, and bile-acid signaling. Through these interconnected layers, BBR coordinates glucose and lipid metabolism while modulating inflammatory tone [74,108,109].

This network pharmacology provides a robust foundation for rational combination therapy. BBR demonstrates strong combination potential. When paired with agents acting through orthogonal mechanisms—such as prebiotics, anti-inflammatory drugs, or bile-acid modulators—additive or synergistic improvements in glycemia, adiposity, hepatic steatosis, and inflammatory markers have been consistently observed [110,111,112]. This positions BBR not merely as a monotherapy, but as a versatile platform for building enhanced therapeutic regimens.

The emerging data suggest that BBR may induce durable metabolic remodeling. Certain benefits, such as improved glucose homeostasis and microbiota composition, appear to persist beyond treatment withdrawal, potentially mediated by sustained epigenetic modifications and a stabilized microbial ecosystem [113]. While promising, the causal mechanisms and long-term clinical durability of these “post-treatment effects” require rigorous validation through controlled, longitudinal studies. Future research priorities span mechanistic, computational, and translational domains. Mechanistically, efforts should focus on causal resolution—using stable-isotope tracing to map microbially derived metabolites and their host routing [113], spatial multi-omics or gut-on-a-chip systems to delineate crypt–villus pharmacology [114,115], and targeted validation of epigenetic or receptor-dependent pathways. Computationally, AI-assisted decision frameworks integrating microbiome, metabolome, and clinical datasets could enable precision responder identification, rational combination design, and adaptive dose optimization, provided that model governance and external validation are in place [116,117,118,119]. Translationally, overcoming bioavailability and safety bottlenecks remains essential. Poor oral absorption, CYP450 inhibition, and gastrointestinal intolerance can be mitigated through formulation and chemistry innovations, such as self-emulsifying systems [120,121,122,123], structure–activity optimization to reduce interaction liabilities, and colon-targeted chitosan microspheres to enhance local tolerability [124,125,126].

However, BBR’s clinical utility has been limited primarily by its low bioavailability [127], which is influenced by several key factors. One of the main reasons for BBR’s poor absorption is that it is a substrate for P-glycoprotein (P-gp) in the intestine, which actively effluxes BBR back into the intestinal lumen, thereby reducing its absorption. Additionally, BBR tends to accumulate in the intestinal tract, which further limits its systemic bioavailability. After oral administration, BBR undergoes extensive first-pass metabolism in the liver, which reduces its concentration before it reaches the bloodstream. Furthermore, BBR is rapidly excreted via bile and urine, limiting its duration of action in the body. These factors underscore the need for effective formulation strategies to improve BBR’s absorption, extend its systemic presence, and enhance its therapeutic efficacy [128].

Recent advancements in BBR research have focused on enhancing its bioavailability, particularly through combination therapies and novel drug delivery systems. In combination therapies, interactions between BBR and other drugs or excipients can alter its pharmacokinetics, leading to improved absorption, altered metabolism, and greater therapeutic efficacy. One strategy involves the use of absorption enhancers such as tetrandrine, which has been shown to improve BBR’s intestinal absorption by inhibiting P-glycoprotein (P-gp), a transporter that restricts drug absorption. This approach may increase BBR’s levels in the blood and improve its therapeutic efficacy [127,129]. Another approach involves co-administration with natural compounds like Curcumin or Ginseng, which not only have complementary pharmacodynamic effects but also modify BBR’s pharmacokinetics. For instance, Curcumin inhibits CYP3A4, the enzyme responsible for BBR’s first-pass metabolism, potentially increasing its bioavailability [130,131]. Additionally, nanocarriers such as liposomes or polymeric nanoparticles have been explored to improve the solubility, stability, and absorption of BBR. These systems protect BBR from metabolic degradation and enable sustained release, maintaining effective drug concentrations over time [132,133]. Self-assembled nanoparticles or micelles, created by combining BBR with amphiphilic molecules, further enhance solubility in aqueous environments. This self-assembly process improves BBR’s stability, solubility, and cellular uptake, providing targeted delivery to tissues such as the liver, where BBR exerts its therapeutic effects. Self-assembly also enables controlled and sustained release, optimizing BBR’s pharmacokinetics [110,133].

The improvement in BBR’s bioavailability through these strategies could explain, at least in part, the synergistic effects observed when BBR is combined with other drugs or natural compounds. Studies have shown that when BBR is used in conjunction with other agents, the therapeutic effects are enhanced beyond what would be expected from either agent alone. This is likely due to improved pharmacokinetics and a greater effective concentration of BBR achieved through the combination. For example, in the treatment of Type 2 Diabetes Mellitus (T2DM), combining BBR with Metformin may lead to more pronounced effects on insulin sensitivity and glucose metabolism, resulting in better blood glucose control [76,124]. Similarly, when combined with Statins for cardiovascular diseases, BBR may enhance their lipid-lowering effects, especially when its bioavailability is improved through delivery systems or absorption enhancers [110].

While significant progress has been made in enhancing BBR’s bioavailability, further research is needed to explore new drug delivery technologies that can target BBR more effectively to specific tissues, such as the liver, where it exerts much of its therapeutic action. Investigating individual variations in bioavailability is also crucial, as genetic or environmental factors may influence BBR’s absorption and metabolism in different patients. Additionally, clinical studies are needed to validate improvements in bioavailability and their direct correlation with enhanced clinical outcomes, particularly in combination therapies.

The European Pharmacopeia (Ph. Eur.) monograph for BBR establishes definitive criteria for quality control, purity assessment, and analytical validation, ensuring pharmaceutical consistency and safety. It specifies critical attributes, including structural identity, identification methods, and pharmacopeial-grade purity standards. For quantification, reversed-phase HPLC with UV detection at 345 nm is mandated, using a defined mobile phase (e.g., acetonitrile–0.1% phosphoric acid) to achieve precise assay accuracy (±2%). Impurity limits are rigorously enforced (individual impurities ≤ 0.5%, total ≤ 1.0%), with validated methods for detecting heavy metals (≤20 ppm), residual solvents, and moisture (≤5.0% by Karl Fischer). Stability parameters, such as pH range, loss on drying, and storage conditions (airtight containers, 15–25 °C), pre-serve integrity during shelf life. These specifications underpin therapeutic efficacy and regulatory compliance, serving as a global benchmark for manufacturing and quality assurance.

In conclusion, within the “hub–axis” paradigm presented herein, BBR should be reframed from a traditional multi-target nutraceutical into a prototype system-level metabolic modulator. Its greatest value may lie as a pharmacological scaffold that bridges deep mechanistic insight with practical clinical translation. Future success will depend on biomarker-guided trial designs, the use of mechanism-anchored endpoints (e.g., MRI-PDFF, bile-acid/SCFA profiles), and adequately powered randomized trials that quantify its durable benefits, safety, and dose-sparing potential in rational combinations. Ultimately, BBR exemplifies a path forward for managing cardiometabolic complexity—by rationally modulating the network, rather than merely inhibiting a single node.

## Figures and Tables

**Table 1:** Drug combinations of BBR.

Drug Combination	Dosage of API	Interaction of API	Combined Mechanism	Synergistic Pharmacodynamics	Research Status
BBR+UDCA	BBR+UDCA (1000 mg) [70]	Compared with using BBR alone and UDCA alone, BUDCA can increase the half-life of the drugs, and its PK profile is better than the alternative [71]	FXR, AMPK, inflammatory axis	Systemic metabolic-inflammatory co-regulation	Phase II clinical trials, clear mechanism [70,71,72,73]
BBR + Metformin	Met (250 mg/kg) BBR (125 mg/kg) [74]	A pharmacokinetic interaction caused by cooperatively inhibiting OCT and MATE1-mediated transport [75]	AMPK stacking, microecological regulation	Enhanced hypoglycemic effect and improved tolerance	Supported by both clinical and animal studies [74,76,77]
BBR+ Prebiotics/Postbiotics	BBR (0.6 g per 6 pills, twice daily before meal) probiotics (4 g per 2 strips of powder, once daily at bedtime) [78]	Potentially improves BBR bioavailability [79,80]	Microecological restoration, immune regulation	Negative entropy effect of the gut–liver axis	Hotspots in preclinical research [78]
BBR + Anti-inflammatory drugs	BHS: BBR (6): hypaconitine (2): skimmianine (1) [81] BBR (20 mg/kg) plus fingolimod (0.15 mg/kg) [82]	Reduces the drug dosage of BBR and improves its bioavailability [82]	COX-2, inflammasome regulation	The prevention and control of anti-MASH and pre-cancer	Preliminary animal data [81,82]
BBR + SGLT2 inhibitors	BBR + SGLT2 inhibitors	No experimental proof	Glucose excretion + energy balance reconstruction	Synergistic improvement of blood glucose and body weight	Supported by theory, under clinical exploration

## Data Availability

No new data were created or analyzed in this study. Data sharing is not applicable to this article.

## Abbreviations

The following abbreviations are used in this manuscript:

BBR	Berberine
AMPK	AMP-activated Protein Kinase
SIRT1/3	Sirtuin 1/3
NF-κb	Nuclear Factor Kappa-light-chain-enhancer of Activated B cells
NLRP3	NOD-, LRR- and Pyrin Domain-containing Protein 3
SGLT2	Sodium-Glucose Cotransporter 2
MASLD	Metabolic Dysfunction-Associated Steatotic Liver Disease
NAFLD	Non-Alcoholic Fatty Liver Disease
InsR	insulin receptor
PI3K	Phosphoinositide 3-Kinase
AKT	Protein Kinase B
mTOR	Mechanistic Target of Rapamycin
GLUT4	Glucose Transporter Type 4
AS160	AKT Substrate of 160 kDa
TBC1D4	TBC1 Domain Family Member 4
TBC1D1	TBC1 Domain Family Member 1
ACC	Acetyl-CoA Carboxylase
SREBP-1c	Sterol Regulatory Element-Binding Protein 1c
CPT1	Carnitine Palmitoyltransferase 1
LKB1	Liver Kinase B1
PGC-1α	Peroxisome Proliferator-Activated Receptor Gamma Coactivator 1-Alpha
TLR4	Toll-like Receptor 4
FXR	Farnesoid X Receptor
TGR5	Takeda G Protein-Coupled Receptor 5
AMP	Adenosine Monophosphate
ADP	Adenosine Diphosphate
STK11	Serine/Threonine Kinase 11
AMPKα1	AMP-activated Protein Kinase α1 subunit
CaMKKβ	Calcium/Calmodulin-Dependent Protein Kinase Kinase Beta
TAK1	Transforming Growth Factor Beta-Activated Kinase 1
NAMPT	Nicotinamide Phosphoribosyltransferase
NAD^+^	Nicotinamide Adenine Dinucleotide
NDUFS1	NADH Dehydrogenase Ubiquinone Fe-S Protein 1
IκB	Inhibitor of κB
IL-1β	Interleukin-1 β
IL-18	Interleukin-18
EIF2AK2	Eukaryotic Translation Initiation Factor 2 Alpha Kinase 2
JNK	c-Jun N-terminal Kinase
TSC2	Tuberous Sclerosis Complex 2
C/EBPα	CCAAT/Enhancer-Binding Protein Alpha
PPARγ	Peroxisome Proliferator-Activated Receptor Gamma
LDLR	Low-Density Lipoprotein Receptor
NADH	Nicotinamide Adenine Dinucleotide + Hydrogen
LPS	Lipopolysaccharide
TMA	Trimethylamine
TMAO	Trimethylamine N-oxide
MUC2	Mucin 2
ERK	Extracellular Regulated Protein Kinase
TGR5	G Protein-Coupled Bile Acid Receptor 5
GLP-1	Glucagon-Like Peptide-1
DNMT1/3a	DNA Methyltransferase 1/3a
TET2	Tet Methylcytosine Dioxygenase 2
HDAC3/4	Histone Deacetylase 3/4
LDL	Low-Density Lipoprotein
LOX-1	Lectin-like Oxidized Low-Density Lipoprotein Receptor-1
ABCA1	ATP-Binding Cassette Subfamily A Member 1
BDNF	Brain-Derived Neurotrophic Factor
FFAR2/3	Free Fatty Acid Receptor 2/3
DPP-4	Dipeptidyl Peptidase-4
UCP1	Uncoupling Protein 1
PYY	Peptide YY
ALT	Alanine Aminotransferase
AST	Aspartate Aminotransferase
JAK	Janus Kinase
STAT	Signal Transducer and Activator of Transcription
CYP450	Cytochrome P450
PCOS	polycystic ovary syndrome
SCFAs	short-chain fatty acids
CPT1A	Carnitine Palmitoyltransferase 1A
H3K9ac	Histone H3 Lysine 9 Acetylation
H3K27me3	Histone H3 Lysine 27 Trimethylation
PTEN	Phosphatase and Tensin Homolog
MRI-PDFF	Magnetic Resonance Imaging Proton Density Fat Fraction
G6PC	Glucose-6-Phosphatase
PCK1	Phosphoenolpyruvate Carboxykinase 1
TGF-β	Transforming Growth Factor-beta
ZO-1	Zonula Occludens-1
TLR4/MyD88	Toll-Like Receptor 4/Myeloid Differentiation Primary Response 88
eNOS	endothelial nitric oxide synthase
Aβ/Tau	Amyloid-beta/Microtubule-Associated Protein Tau
HIV	Human Immunodeficiency Virus
UDCA	ursodeoxycholic acid
Met	metformin
SGLT2i	Sodium-Glucose Cotransporter 2 inhibitor
COX-2	cyclooxygenase-2
BUDCA	BBR ursodeoxycholate
T2DM	Type 2 Diabetes Mellitus
FOS	FBJ Murine Osteosarcoma Viral Oncogene Homolog
IKK	IκB Kinase
MAPK	Mitogen-Activated Protein Kinase
NSAIDs	Non-Steroidal Anti-Inflammatory Drugs
TC	Total Cholesterol
LDL-C	Low-Density Lipoprotein Cholesterol
